# Amelioration of Isoproterenol-Induced Oxidative Damage in Rat Myocardium by *Withania somnifera* Leaf Extract

**DOI:** 10.1155/2015/624159

**Published:** 2015-10-11

**Authors:** Md. Ibrahim Khalil, Istiyak Ahmmed, Romana Ahmed, E. M. Tanvir, Rizwana Afroz, Sudip Paul, Siew Hua Gan, Nadia Alam

**Affiliations:** ^1^Laboratory of Preventive and Integrative Biomedicine, Department of Biochemistry and Molecular Biology, Jahangirnagar University, Savar, Dhaka 1342, Bangladesh; ^2^Human Genome Centre, School of Medical Sciences, Universiti Sains Malaysia, 16150 Kubang Kerian, Kelantan, Malaysia

## Abstract

We investigated the protective role of *Withania somnifera* leaf extract (WSLEt) on isoproterenol- (ISO-) induced myocardial infarction (MI) in rats. Subcutaneous injection of ISO (85 mg/kg body weight (b.w.)) administered to rats for two consecutive days caused a significant increase in cardiac troponin I (cTnI) levels and serum lipid profiles, as well as the activities of some marker enzymes. In addition to these diagnostic markers, there were increased levels of lipid peroxidation (LPO) and decreased activities of enzymatic antioxidants (superoxide dismutase (SOD), glutathione peroxidase (GPx), glutathione reductase (GRx), and glutathione-S-transferase (GST)) in the myocardium. However, oral pretreatment (100 mg/kg b.w.) with WSLEt for 4 weeks elicited a significant cardioprotective activity by lowering the levels of cTnI, lipid profiles, and marker enzymes. The levels of LPO products were also significantly decreased. Elevated activities of antioxidant enzymes were also observed in rats pretreated with WSLEt. As further confirmed histopathologically, our findings strongly suggest that the cardioprotective effect of WSLEt on myocardium experiencing ISO-induced oxidative damage may be due to an augmentation of the endogenous antioxidant system and an inhibition of LPO in the myocardial membrane. We conclude that WSLEt confers some protection against oxidative damage in ISO-induced MI in rats.

## 1. Introduction

*Withania somnifera* (Solanaceae), also known as “ashwagandha” or “winter cherry,” is one of the most valuable herbs in the traditional Indian systems of medicine [1]. The plant is utilized in more than 100 formulations in Ayurveda, Unani, and Siddha [2]. It is described as an herbal tonic and health food in the famous book of Vedas and is considered akin to an “Indian Ginseng” in the traditional Indian system of healing [3]. The ethnopharmacological properties of the plant include adaptogenic, antisedative, and anticonvulsive activities. The plant is used to treat various neurological disorders, geriatric debilities, arthritis, stress, and behavior-related problems [4].* W. somnifera* contains a variety of nutrients and phytochemicals and is therefore also used as a dietary supplement. It has been reported that all of the major parts of* W. somnifera*, such as the roots, fruits, and leaves, provide potential benefits for human health because of their high polyphenol contents and antioxidant activities [5].

Myocardial infarction (MI) is a common presentation of ischemic heart disease (IHD). MI remains the major cause of death in the developed world and is a major pathological issue worldwide despite rapid advancements made in the treatment of coronary artery diseases (CAD) [6]. It occurs as a result of increased myocardial metabolic demand and decreased supply of oxygen and nutrients via the coronary circulation to the myocardium, leading to cell injury; it is one of the most lethal manifestations of cardiovascular diseases (CVD) [7]. MI continues to be a major public health problem, not only in western countries but also increasingly more in developing countries, where it contributes significantly to mortality [8]. According to the World Health Organization, MI is predicted to be the major cause of death in the world by the year of 2020 [9].

Isoproterenol [1–(3, 4–dihydroxyphenyl)–2–isopropylaminoethanol hydrochloride (ISO)] is a synthetic catecholamine and *β*-adrenergic agonist that is an important regulator of myocardial contractility and metabolism, thus serving as the key element of a standard model for the study of potentially beneficial effects of numerous drugs on cardiac function [7]. ISO induces cardiac necrosis by several mechanisms, including increased oxygen consumption, poor oxygen utilization, increased calcium overload and accumulation, altered myocardial cell metabolism, increased myocardial cAMP levels, deranged electrolyte milieu, altered membrane permeability, intracellular acidosis, and increased levels of lipid peroxides [10]. The pathophysiological and morphological aberrations produced in the heart of the myocardial necrotic rat model are comparable with those taking place in human MI. The various mechanisms proposed to explain ISO-induced cardiotoxicity include the generation of highly cytotoxic free radicals through the autooxidation of catecholamines, which has been implicated as one of the important causative factors [11].

Oxidation of catecholamine forms quinoid compounds giving rise to the production of superoxide anions and, subsequently, hydrogen peroxide, which, in the presence of iron, forms highly reactive hydroxyl radicals and causes protein, lipid, and DNA damage and increased MI size [12]. In addition, excessive formation of free radicals may result in the loss of function and integrity of myocardial membranes [13]. These free radicals may attack polyunsaturated fatty acids (PUFAs) within the membranes, forming peroxyl radicals. These radicals can then attack adjacent fatty acids, causing a chain reaction of lipid peroxidation (LPO). The lipid hydroperoxide end products are also harmful and may contribute to further tissue and organ damage [14].

In recent years, long-term prevention of CVD is associated with the consumption of fresh fruits, vegetables, or plants rich in natural antioxidants. As a result, there has been considerable interest in research on natural bioactive compounds, with a generally accepted view that natural products are superior in terms of efficacy and safety when compared to their synthetic analogs [15]. Medicinal plants constitute an important source of active natural products that differ widely in terms of structure and biological properties and play an important role in the protection against various human diseases including CVD [16].

A previous study reported that* W. somnifera*, particularly its leaves, has remarkable antioxidant properties [1].* W. somnifera* leaves have been reported to contain higher amounts of polyphenols and flavonoids when compared to the roots and fruits [5]. To date, many epidemiological studies have demonstrated the effectiveness of phenolics and flavonoids as antitumor, anti-inflammatory agent or in reducing the risk of cardiovascular diseases [17]. Most importantly,* W. somnifera* leaves contain higher levels of catechin, which belongs to the flavonoid family, when compared to other parts of the plant [5]. Some previous studies have strongly suggested that catechin reduces the risk of IHD [18, 19]. To date, there is no or little available data on the potential medicinal properties of* W. somnifera* leaves, since most studies tend to focus on* W. somnifera* roots [7, 20]. Considering the more robust antioxidant potential and higher catechin content of* W. somnifera* leaves, we were interested in investigating the effect of* W. somnifera* leaves on oxidative stress-induced cardiac injury. In this study, the cardioprotective effect of* W. somnifera* leaf extract (WSLEt) was investigated in relation to cardiac marker enzymes, lipid peroxides, and the antioxidant enzyme defense system.

## 2. Materials and Methods

### 2.1. Experimental Animals

The experiments were conducted according to ethical guidelines as approved by the Bangladesh Association for Laboratory Animal Science. Adult male Wistar Albino rats (*n* = 40) (140–160 g) were bred and reared in the animal house facility of the Department of Biochemistry and Molecular Biology, Jahangirnagar University, at a constant room temperature of 23 ± 2°C, and in an environment with humidity ranging between 40% and 70%. The rats were housed in plastic cages (with hard wood chips for bedding) and received a natural 12 h day-night cycle. The rats were provided with a standard laboratory pellet diet and water* ad libitum*. The pellet diet consisted of 56.17% carbohydrate, 22.02% crude protein, 4.25% crude oil, 3.25% crude fibre, 2.46% glucose, 0.8% calcium, 0.6% phosphorus, and 1.8% vitamins.

### 2.2. Drugs and Chemicals

The assay kit used for the estimation of cardiac troponin I (cTnI) levels was purchased from JAJ International, Inc., USA. Other assay kits for the measurement of creatine kinase (CK-MB), lactate dehydrogenase (LDH), aspartate transaminase (AST) and alanine transaminase (ALT), TC (total cholesterol), TGs (triglycerides), and high-density lipoprotein-cholesterol (HDL-C) were all purchased from Stanbio Laboratory, USA. The assay kits for superoxide dismutase (SOD), glutathione peroxidase (GPx), glutathione reductase (GRx), and glutathione-S-transferase (GST) were purchased from Abnova Corporation, Taiwan. ISO and 1,1,3,3-tetraethoxy propane were purchased from Nacalai Tesque, Inc., Kyoto, Japan. All of the chemicals and reagents used in this study were of analytical grade.

### 2.3. Sample Collection and Extraction

*W. somnifera* leaves were collected from the Gaibanda Samriddhi Project, HELVETAS Swiss Inter Cooperation-Bangladesh, in July, 2013, and were authenticated by a botanist (Professor M. Shah Alam, Department of Botany, Rajshahi University, Rajshahi 6205, Bangladesh). The collected leaves of the medicinal plant were cleaned and then air-dried in the shade for 7 days before being ground to a fine powder by a blender (CM/L7360065, Jaipan, Mumbai, India). The fine powder was used to prepare a 5% ethanolic extract (5 g of* W. somnifera* leaf powder added to a final volume of 100 mL of a 70% ethanol solution) in the dark so as to avoid reactions in solution that may occur in the presence of light. The solution was shaken in a shaker for 72 h at room temperature. Then, the solution was filtered and dried in a rotary evaporator (Buchi, Tokyo, Japan) under reduced pressure (100 psi) at a controlled temperature (40°C). The dried extract was collected and then finally preserved at −20°C for subsequent* in vivo* studies. Only the required amount was withdrawn from refrigerator to ensure the stability of the extract.

### 2.4. Experimental Design

**Figure 1:**
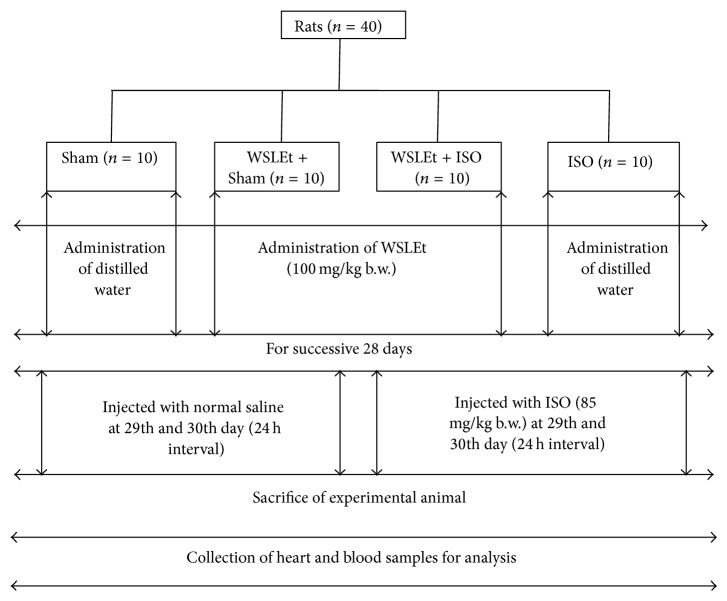
Schematic representation of experimental design of the study.

After a week-long acclimation period, the animals were randomly divided into 4 groups (10 rats in each group) (Figure 1).

*Sham*. Animals received only distilled water (2 mL/kg) for 4 weeks and were then treated by normal saline injection (1 mL) for 2 days (on the 29th and 30th days).

*WSLEt + Sham*. Animals were pretreated with WSLEt (100 mg/kg) for 4 weeks at 24 hr interval and then treated by normal saline injection (1 mL) for 2 days (on the 29th and 30th days).

*WSLEt + ISO*. Animals were pretreated with WSLEt (100 mg/kg) for 4 weeks at 24 hr interval and then treated by ISO injection (85 mg/kg) for 2 days (on the 29th and 30th days).

*ISO*. Animals received only distilled water (2 mL/kg) for 4 weeks and were then treated by ISO injection (85 mg/kg) for 2 days (on the 29th and 30th days).

### 2.5. Induction of Experimental MI

ISO (85 mg) was dissolved in normal saline (1 mL) and was subcutaneously (s.c.) injected into rats (85 mg/kg) at an interval of 24 h for 2 days to induce experimental MI. The choice of ISO dose was based on a pilot study for ISO dose fixation and on the results of a previous study [21]. The WSLEt dosage was based on previous studies [7, 22].

During the experimental period, the rats' body weights were recorded regularly and the doses were modulated accordingly. At 48 h after the first ISO injection, all animals were sacrificed by decapitation. Blood samples (3 mL) were collected and serum samples were separated by centrifugation. Immediately following blood collection, the heart samples were separated from surrounding tissues and were washed twice with ice cold phosphate-buffered saline. The samples were then homogenized in phosphate buffer (25 mM, pH 7.4) using a tissue homogenizer (F 12520121, Omni International, Kennesaw, USA) to produce an approximately 10% w/v homogenate. The homogenate was centrifuged at 1,700 rpm for 10 min, and the supernatant was collected and stored at −20°C for subsequent biochemical analyses. Some of the heart samples were stored in 10% formalin for histopathological examination.

### 2.6. Serum Biochemical Analysis

An enzyme immunoassay kit was employed for the determination of cTnI in serum samples using an ELISA micro-plate reader (digital and analog system RS232, Das, Italy). Standard assay kits were employed to determine the levels of CK-MB, LDH, AST, ALT, TC, TGs, and HDL-C in serum samples using a PD-303S Spectrophotometer (APEL, Japan). Serum VLDL-C levels were calculated based on a formula provided by Friedewald [23]:

(1) $$\begin{array}{c} \text{VLDL-C}=\frac{\text{TG}}{5}. \end{array}$$

### 2.7. Biochemical Analysis in Heart Tissue

Malondialdehyde (MDA) levels were assayed for LPO products in the heart tissues. MDA, which is also referred to as thiobarbituric acid-reactive substance (TBARS), was measured according to the method published by Ohkawa et al. [24]. Briefly, 0.2 mL of tissue homogenate, 0.2 mL of 8.1% sodium dodecyl sulphate (SDS), 1.5 mL of 20% acetic acid, and 1.5 mL of 8% TBA were mixed. The mixture was supplemented up to 4 mL with distilled water and was heated at 95°C in a water bath for 60 min. After incubation, the tubes were cooled to room temperature and the final volume was increased to 5 mL in each tube. A butanol : pyridine (15 : 1) mixture (5 mL) was added and the contents were vortexed thoroughly for 2 min. After centrifugation (3,000 rpm) for 10 min, the upper organic layer was aspirated, and its absorbance was read at 532 nm against the blank. The levels of TBARS were expressed as nmol of MDA per mg of protein.

The heart tissue homogenate was recentrifuged at 12,000 rpm for 10 min at 4°C using an Eppendorf 5415D centrifuge (Hamburg, Germany). The resulting clean supernatants of heart tissue extracts were used for further estimation of endogenous antioxidant enzymes including SOD, GPx, GRx, and GST using standard ELISA micro-plate assay kits. The levels of SOD, GPx, GRx, and GST were expressed as units/mg of protein, nmol of NADPH oxidized/min/mg of protein, nmol of NADPH oxidized/min/mg of protein, and nmol of CDNB conjugated/min/mg of protein, respectively. The total protein in the heart tissue homogenates was estimated by the method described by Lowry et al. [25]. Briefly, 0.2 mL sample (digested with 0.1 N sodium hydroxide (NaOH)) was mixed with 2 mL of working reagent (a mixture of 2% sodium carbonate, 0.1 N NaOH, 1.56% copper sulphate, and 2.37% sodium-potassium tartrate), and the reaction mixture was incubated for 10 min at room temperature. The addition of 1 N Folin-Ciocalteu's phenol reagent (0.2 mL) was followed by a 30 min incubation at room temperature. Finally, the absorbance was measured at 660 nm. Bovine serum albumin was used as the standard to calculate the protein content of samples.

### 2.8. Histopathological Examination

After sacrificing the animals, the hearts were rapidly dissected and immediately washed with saline before being fixed in 10% formalin. The fixed tissues were then embedded in paraffin. After that, serial sections (5 *µ*m thickness) were cut followed by staining with hematoxylin and eosin (H & E). Microscopic observation was done using a fluorescence microscope over normal spectra (Olympus DP72, Tokyo, Japan) at 40x magnification. Photomicrographs were taken by using an attached digital camera. A dedicated pathologist who was blind to the treatment assignment of the different study groups was assigned to perform the histopathological evaluation.

### 2.9. Statistical Analysis

The results of all the groups are shown as mean values ± standard deviations (SD). The data was analyzed using SPSS (Statistical Packages for Social Science, version 20.0, IBM Corporation, New York, USA) and Microsoft Excel 2007 (Redmond, Washington, USA). Statistical analyses of biochemical data were performed using a one-way ANOVA followed by a Tukey* post hoc *test. A *p* value of <0.05 was accepted as indicating statistical significance.

## 3. Results

None of the rats died in any of the experimental groups over the entire 4-week treatment period. There was no significant difference in the body weights observed at the baseline time point or at the end of the experimental period between the groups (Table 1). However, the heart weights increased significantly (*p* < 0.05) in ISO-treated rats when compared with normal control rats. When compared to ISO group, rats pretreated with WSLEt had a significant (*p* < 0.05) reduction in heart weight, indicating its cardioprotective effects. No significant difference was observed in rats treated with WSLEt alone when compared to normal control rats.

**Figure 2:**
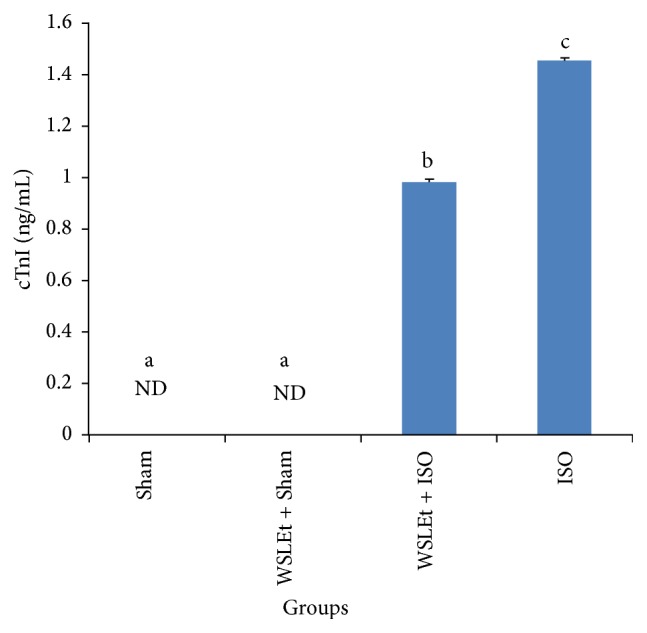
WSLEt ameliorates the oxidative damage caused by ISO as demonstrated by the changes in cTnI levels. Bars represent mean values ± SD (*n* = 10); bars with different letters represent significantly different mean values at *p* < 0.05. ND: not detected.

Rats treated with ISO alone had a marked (*p* < 0.05) elevation in serum cTnI levels when compared to the control (Figure 2). However, oral pretreatment of WSLEt for 4 weeks significantly (*p* < 0.05) decreased serum cTnI levels in ISO-treated rats when compared with ISO group.

**Figure 3:**
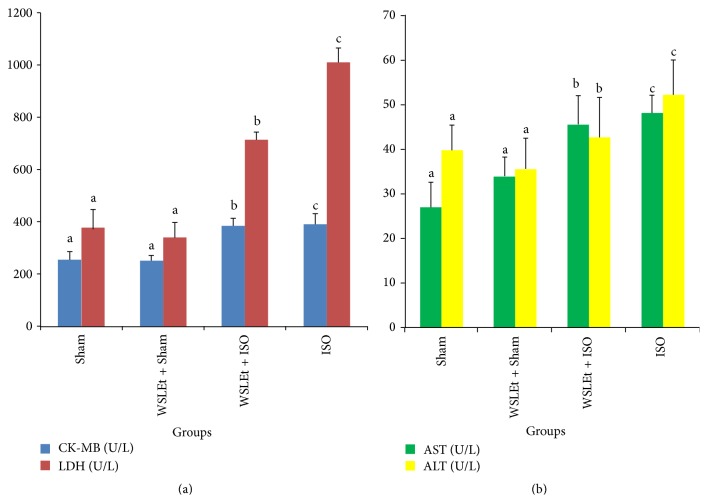
WSLEt ameliorates the oxidative damage caused by ISO as demonstrated by the changes in the cardiac marker enzyme activities. (a) The effects of WSLEt on CK-MB and LDH levels and (b) the effects of WSLEt on AST and ALT levels. Bars represent mean values ± SD (*n* = 10); bars with different letters represent significantly different mean values at *p* < 0.05.

A marked increase in the activities of serum cardiac enzymes was observed in ISO-induced myocardial ischemic rats (Figure 3). This effect was significantly ameliorated by WSLEt.

Similarly, pretreatment with WSLEt also ameliorated serum lipid profile increases (TC, TGs, VLDL-C, and HDL-C) (Table 2).

The activities of antioxidant enzymes such as SOD, GRx, GPx, GST, and LPO in the hearts of ISO-treated rats, which were significantly decreased when compared with the control, improved significantly by pretreatment with WSLEt (Table 3).

**Figure 4:**
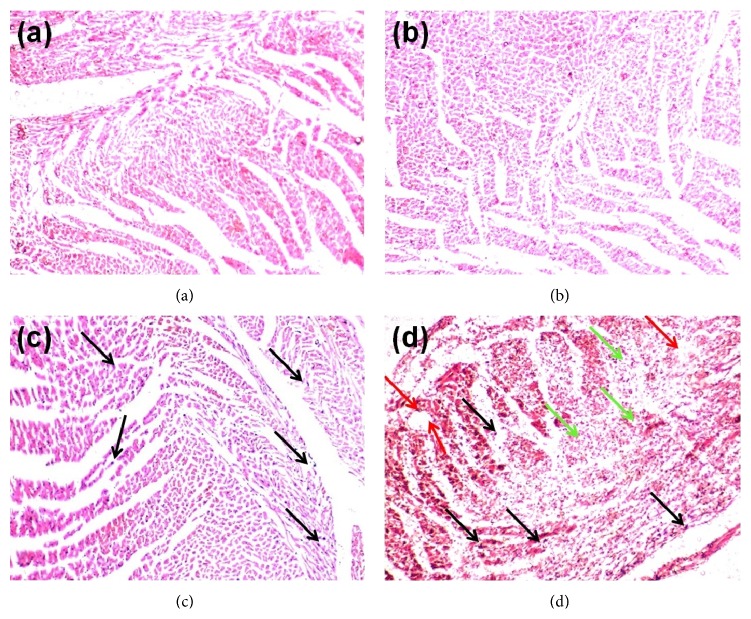
(a) Sham group: normal control heart showing normal cardiac muscle fibers. (b) WSLEt + Sham group: WSLEt-treated (100 mg/kg) heart showing normal muscle fibers without any pathological changes. (c) WSLEt + ISO group: WSLEt-treated (100 mg/kg) + ISO-treated (85 mg/kg) heart showing no edematous intramuscular space and fewer inflammatory cells (black arrows). (d) ISO group: ISO-treated (85 mg/kg) heart showing cardiac muscle fibers with muscle separation (green arrows), edematous intramuscular space (red arrows), and inflammatory cells (black arrows).

Figures 4(a)–4(d) show the effects of WSLEt on the histology of the heart in normal and ISO-induced myocardial-infarcted rats. Control rats and those treated with WSLEt (100 mg/kg) showed normal cardiac fibers (Figures 4(a) and 4(b)) with no overt damage observed.  Figure 4(d) shows an ISO-treated myocardium with an area of infarction with splitting of cardiac muscle fibers, edematous intramuscular space, and inflammatory cells. Animals from WSLEt + ISO, however, had cardiac muscle fibers with significantly fewer inflammatory cells (Figure 4(c)).

## 4. Discussion

To our knowledge, our study is the first to demonstrate the cardioprotective effect of WSLEt. The experimental animal hearts revealed a significant increase in both their absolute and relative weights following ISO administration, although the body weight remained relatively unchanged. The increase in heart weight may be due to increased water accumulation with edematous intramuscular spaces in heart tissue and increased protein content [10], which is also confirmed by the histopathological findings. It has been proposed that myocardial function may be reduced by approximately 10% due to an increase in myocardial water content by 1% [26]. Increased membrane permeability in the pathogenesis of cardiac muscle cell injury following catecholamine toxicity is purported to be one of the main contributing factors to water accumulation in the heart [11]. Catecholamines are important regulators of myocardial contractility and metabolism. However, it has been long known that excess levels of catecholamines are responsible for cellular damage, as observed in clinical conditions such as angina, transient myocardial hypoxia, acute coronary insufficiency, and subendocardial infarct. The administration of ISO has effects on mitochondrial LPO, antioxidants, TCA cycle enzymes, and respiratory marker enzymes. Animals develop infarct-like lesions when injected with ISO, a potent synthetic catecholamine [27]. Increased generation of cytotoxic free radicals as a result of the autooxidation metabolic products of ISO is one of the well-recognized mechanisms of ISO-induced myocardial necrosis [28]. Pretreatment with WSLEt, however, significantly decreased the absolute and relative heart weights, bringing them close to their normal values, which indicates the protective effect of the WSLEt on the myocardium against infiltration or accumulation with water.

Cardiac troponin is a low molecular weight protein which is a constituent of the myofibrillary contractile apparatus of the cardiac muscle. cTnI has been shown to be a highly sensitive and specific marker of myocardial cell injury; it is usually absent in serum in normal individuals and released only after myocardial necrosis [29]. In this study, an increased level of serum cTnI in ISO-treated rats was observed relative to the control group. The increased level of cTnI may be attributed to the ISO-induced cardiac damage. Animals treated with ISO following pretreatment with WSLEt, however, exhibited a significant reduction in cTnI levels when compared to ISO-treated rats without the WSLEt pretreatment. Our results are consistent with those from a previous study reported by Priscilla and Prince [14]. Pretreatment with WSLEt significantly decreased serum cTnI levels in ISO-treated cardiotoxic rats. It is assumed that WSLEt may preserve the structural and functional integrity of the contractile apparatus, which prevents cardiac damage and leakage of troponins from the heart into the blood. Nevertheless, further research is essential to elucidate the exact mechanisms underlying the cardioprotective effect of WSLEt.

The myocardium contains high concentrations of diagnostic markers of MI; once it is metabolically damaged, it releases its contents into the extracellular fluids [30]. Of all the macromolecules leaked from the damaged tissue, myocardial enzymes are the best markers of tissue damage because of their tissue specificity and catalytic activity. When myocardial cells are damaged or destroyed due to a deficiency in the oxygen supply or glucose, the cardiac membrane becomes permeable or may rupture entirely, resulting in the leakage of enzymes [14]. The activity assay for CK-MB in serum is an important diagnosis because of the marked abundance of this enzyme in myocardial tissue and its virtual absence from most other tissues and its consequent sensitivity. CK-MB isoenzyme activity is useful as an index for the early diagnosis of not only myocardial infarction, but also any type of myocardial injury. Leakage of cytosolic enzymes including CK-MB, LDH, AST, and ALT (which serve as diagnostic markers from the damaged tissue) into the blood stream may occur when cell membranes become more permeable or rupture. The amounts of these cellular enzymes in the serum reflect the alterations in plasma membrane integrity and/or permeability [8]. Furthermore, the amount of the enzymes appearing in serum is reported to be proportional to the number of necrotic cells [31], which also reflects a nonspecific alteration in the plasma membrane integrity and/or permeability as a response to *β*-adrenergic stimulation [16]. In the present study, rats administered with ISO showed significant increases in the levels of all these marker enzymes in serum, in line with the results from previous reports, indicating ISO-induced necrotic damage of the myocardium and leakiness of the plasma membrane [14, 22, 32]. Pretreatment with WSLEt, however, resulted in lowered activities of all marker enzymes in the serum, indicating that WSLEt helps in maintaining the membrane integrity, thereby restricting the leakage of these enzymes. Phenolic acids such as gallic acid, syringic acid, vanillic acid, and p-coumaric acid and flavonoids such as catechin and naringenin are important constitutive antioxidants found in WSLEt, as in our study and that reported by Alam et al. [5]. Tanvir et al. [33] and Afroz et al. [34] speculated that antioxidant compounds present in their sample confer protective effects on liver by preserving the membrane integrity. Therefore, it is plausible that the presence of these antioxidants may help protect against oxidative cardiac injury, thus restricting the leakage of these enzymes from the myocardium. For instance, Arts et al. [18] evaluated the effects of catechin intake on the health risks of high levels of body fat and the incidence of IHD and stroke in a cohort of elderly men; according to the study, catechin, whether from tea or other sources, may reduce the risk of IHD mortality. It is suggested that flavonoids decrease the risk of CDV by improving coronary vasodilation, decreasing the ability of platelets in the blood to clot, and preventing low-density lipoproteins from oxidizing [35].

Lipids play an important role in CVD, not only by contributing to the development of atherosclerosis but also by modifying the composition, structure, and stability of the cellular membrane. High levels of circulating cholesterol and its accumulation in heart tissue have been associated with cardiovascular damage [36]. Rats treated with ISO showed a significant increase in serum levels of TC, TGs, and VLDL-C, as previously reported [37]. Generally, the mechanism of actions of lipolytic hormones, including ISO, on fat cells are believed to be mediated by the cAMP cascade, in which lipolytic hormones activate adenylate cyclase, thereby increasing cAMP formation. Subsequently, cAMP promotes lipolytic activity by activating cAMP-dependent protein kinase, which phosphorylates hormone-sensitive lipase [38]. This results in the hydrolysis of stored triacylglycerol, which may contribute to hyperlipidemia [39]. High levels of LDL-C and VLDL-C have been positively correlated with MI but are negatively correlated with HDL-C. HDL-C inhibits the uptake of LDL-C by the arterial walls and facilitates the transport of cholesterol from peripheral tissues to the liver, where it is catabolized and excreted from the body [40]. Pretreatment with WSLEt, however, significantly ameliorates these changes, thereby maintaining the normal fluidity and function of the myocardial membrane. Polyphenols, particularly gallic acid and catechin, have been reported to inhibit cholesterol esterase [41]. In general, pancreatic cholesterol esterase plays an important role in hydrolyzing dietary cholesterol esters, which liberates free cholesterol in the lumen of the small intestine [42]. Therefore, the inhibition of cholesterol esterase is expected to limit the absorbance of dietary cholesterol, resulting in reduced cholesterol absorption. Moreover, polyphenols can also bind with bile acids to increase their fecal excretion, which has been hypothesized as a possible mechanism for the lowering of plasma cholesterol levels by polyphenols [41].

LPO is a well-established mechanism of cellular injury and has been used as an indicator of oxidative stress that leads to the pathogenesis of MI [7]. The degree of LPO has been evaluated by estimating TBARS, lipid hydroxides, and the presence of conjugated dienes [7]. Lipid peroxide-mediated myocardial damage has been observed in ISO-treated myocardial-infarcted rats. The myocardial necrosis observed in the rats receiving ISO can be attributed to peroxidative damage, as it has been previously reported that ISO generates lipid peroxides [43]. In our study, ISO treatment resulted in a significant increase in the levels of LPO products in the heart tissue. Increased LPO appears to be the initial stage of the pathogenesis making heart tissue more susceptible to oxidative damage. WSLEt pretreatment significantly reduces the levels of lipid peroxides in ISO-treated rats. Thus, it is plausible that some constituents present in WSLEt with antioxidant activities scavenge the LPO products produced excessively by ISO and confer protection to the cardiac tissue.

The oxidative stress may be exerted through quinone metabolites of ISO that react with oxygen to produce superoxide anions and other reactive oxygen species (ROS) that interfere with antioxidant enzymes [19]. The presence of the endogenous antioxidant enzymatic defense is highly important for the neutralization of oxygen-free-radical-mediated tissue injury [44]. SOD, catalase (CAT), and GPx, which are the primary free radical scavenging enzymes, are involved in the first-line cellular defense against oxidative injury, decomposing oxygen (O_2_) and hydrogen peroxide (H_2_O_2_) before their interaction to form the more reactive hydroxyl radical [45]. In this study, significantly lower activities of SOD and GPx were observed in the heart tissues of ISO-treated rats when compared to control rats. The observed decreases in the activities of these enzymes may be due to their increased utilization for scavenging ROS and their inactivation by excessive ISO oxidation [16]. Treatment with WSLEt, however, improved the activities of SOD and GPx by scavenging superoxide and H_2_O_2_ produced by ISO. The two enzyme levels were also higher in WSLEt alone treated group when compared with the control group which is a clear indication that WSLEt not only scavenges the oxidative stress but also boosts the activity of few antioxidant enzymes during normal physiological conditions. GRx is an antioxidant enzyme involved in the reduction of GSSG (an end product of the GPx reaction) to GSH [21].

In ISO-treated rats, there was a marked reduction in GPx activity, leading to a reduced availability of substrate for GRx, thereby decreasing its activity. Oral treatment with WSLEt in ISO-treated rats restored the activity of GRx, which accelerates the conversion of GSSG to GSH. A phase II enzyme such as GST not only catalyzes the conjugation of both hydroquinones and epoxides of polycyclic aromatic hydrocarbons with GSH for their excretion, but also shows lower activity towards organic hydroperoxides for their detoxification from cells/tissues [19]. In ISO-treated rats, there was a marked reduction in GST activity, but the activity of this phase II enzyme was restored in WSLEt-treated rats. More interestingly GST levels doubled in WSLEt alone treated group; that is, WSLEt can show a strong potential to enhance GST activity in healthy individuals. It is plausible that the upregulation of the activity or expression of Nrf2, a transcription factor released from its repressor (Keap1) under oxidative or xenobiotic stress [46], is considered as possible mechanism through which WSLEt pretreatment restores antioxidant enzyme functions as also suggested by Erejuwa et al., 2011 [47]. The released Nrf2 binds to the antioxidant response element of cytoprotective genes and induces their expression as well as the subsequent expression of free radical scavenging enzymes to neutralize and eliminate the cytotoxic oxidants [46].

A histopathological examination of the myocardial tissue of normal control rats clearly illustrated the integrity of the myocardial cell membrane. The histopathology of the WSLEt-pretreated myocardial-infarcted heart samples showed a near normal morphology of cardiac muscle with the absence of necrosis when compared to ISO-treated samples without WSLEt pretreatment, which further confirms the biochemical findings. Similar histopathological findings were observed in ISO-treated rats for gallic acid [14], which also has strong antioxidant properties. Overall, the results of this study offer scientific evidence of the importance of WSLEt in cardioprotection against CVD, a set of diseases whose pathogenesis has long been associated with oxidative stress. Further studies should be conducted to elucidate the exact mechanism of the cardioprotective effect of WSLEt.

## 5. Conclusion

The present biochemical and histopathological findings confirm that WSLEt preserves the integrity of myocardial cell membrane by maintaining the activities of cTnI and marker enzymes in the serum and heart of ISO-treated cardiotoxic rats. This may be due to the antilipoperoxidative and antioxidant effects of WSLEt. We conclude that* W. somnifera* leaves have the potential to be used as cardioprotective agents by protecting cardiac tissue against oxidative damage.

## Figures and Tables

**Table 1:** Changes in the body and heart weights in different groups of rats.

Parameters	Group			
	Sham	WSLEt + Sham	WSLEt + ISO	ISO
Initial body weight (g)	143.70 ± 24.16^a^	146.71 ± 15.99^a^	141.00 ± 23.43^a^	148.63 ± 19.91^a^
Final body weight (g)	164.82 ± 19.28^a^	175.00 ± 6.36^a^	169.75 ± 16.64^a^	172.00 ± 21.59^a^
Body weight gain (g)	21.12^a^	28.29^a^	28.75^a^	23.37^a^
Absolute heart weight (g)	0.62 ± 0.03^a^	0.66 ± 0.03^a^	0.73 ± 0.02^b^	0.97 ± 0.06^c^
Relative heart weight (g/100 g)	0.39 ± 0.02^a^	0.39 ± 0.01^a^	0.49 ± 0.02^b^	0.57 ± 0.04^c^

Results are expressed as mean values ± SD; *n* = 10. ^a,b,c^Values in the same row that do not share superscript letters (a, b, c) differ significantly at *p* < 0.05.

**Table 2:** WSLEt ameliorates the oxidative damage caused by ISO as demonstrated by the changes in the serum lipid profiles.

Parameters	Group			
	Sham	WSLEt + Sham	WSLEt + ISO	ISO
TC (mg/dL)	50.19 ± 6.34^a^	45.67 ± 4.93^a^	56.37 ± 6.38^b^	74.75 ± 12.35^c^
TG (mg/dL)	42.69 ± 5.55^a^	38.46 ± 8.09^a^	67.56 ± 8.90^b^	82.29 ± 6.12^c^
VLDL-C (mg/dL)	8.54 ± 1.11^a^	7.69 ± 1.39^a^	12.33 ± 1.41^b^	16.46 ± 1.22^c^
HDL-C (mg/dL)	45.12 ± 2.71^a^	49.12 ± 4.49^b^	46.04 ± 1.15^ab^	19.89 ± 1.21^c^

Results are expressed as mean values ± SD; *n* = 10. ^a,b,c^Values in the same row that do not share superscript letters (a, b, c) differ significantly at *p* < 0.05.

**Table 3:** WSLEt ameliorates the oxidative damage caused by ISO as demonstrated by the changes in LPO levels and the activities of SOD, GRx, GPx, and GST.

Parameters	Group			
	Sham	WSLEt + Sham	WSLEt + ISO	ISO
LPO (nmol TBARS/mg of protein)	42.77 ± 1.05^a^	37.18 ± 1.85^a^	40.02 ± 1.17^a^	82.17 ± 1.35^b^
SOD (units/mg of protein)	1.45 ± 0.02^ab^	1.58 ± 0.16^a^	0.33 ± 0.02^b^	0.10 ± 0.00^c^
GRx (nmol NADPH oxidized/min/mg of protein)	97.56 ± 2.09^a^	90.57 ± 1.79^a^	97.14 ± 6.05^a^	75.59 ± 9.79^b^
GPx (nmol NADPH oxidized/min/mg of protein)	2.15 ± 0.45^a^	3.12 ± 0.16^b^	1.98 ± 0.00^ab^	0.96 ± 0.00^c^
GST (nmol of CDNB conjugated/min/mg of protein)	2.04 ± 0.06^a^	4.05 ± 0.19^b^	1.99 ± 0.11^a^	0.85 ± 0.02^c^

Results are expressed as mean values ± SD; *n* = 10. ^a,b,c^Values in the same row that do not share superscript letters (a, b, c) differ significantly at *p* < 0.05.
