# Expression of Concern on “Amelioration of Isoproterenol‐Induced Oxidative Damage in Rat Myocardium by *Withania Somnifera* Leaf Extract”

**DOI:** 10.1155/bmri/9794680

**Published:** 2026-08-18

**Authors:** BioMed Research International

EXPRESSION OF CONCERN: M. I. Khalil, I. Ahmmed, R. Ahmed, E. M. Tanvir, R. Afroz, S. Paul, S. H. Gan, N. Alam, “Amelioration of Isoproterenol‐Induced Oxidative Damage in Rat Myocardium by Withania somnifera Leaf Extract,” BioMed Research International, 2015, 624159, https://doi.org/10.1155/2015/624159

This Expression of Concern is for the above article, published online on 11 October 2015 in Wiley Online Library (https://onlinelibrary.wiley.com/), and has been issued by agreement between the journal Editor‐in‐Chief, Yoel Klug, and John Wiley & Sons Ltd. The Expression of Concern has been agreed due to concerns identified with Figure 4. Specifically, in Figure 4, panels A and B appear identical to panels A and B in Figure 5 of [1].

Following assessment of the concerns raised, the authors’ response, and the absence of original image data, the publisher was unable to resolve concerns regarding the apparent reuse of image panels across multiple publications from the same research group.

As these concerns could not be satisfactorily addressed, the publisher has issued this Expression of Concern to inform readers.

## References

[1] Afroz R. , Tanvir E. M. , Karim N. , Hossain M. S. , Alam N. , Gan S. H. , and Khalil M. I. , Sundarban Honey Confers Protection against Isoproterenol-Induced Myocardial Infarction in Wistar Rats, BioMed Research International. (2016) 6437641, 10.1155/2016/6437641, 27294126. PMC4886051 27294126

